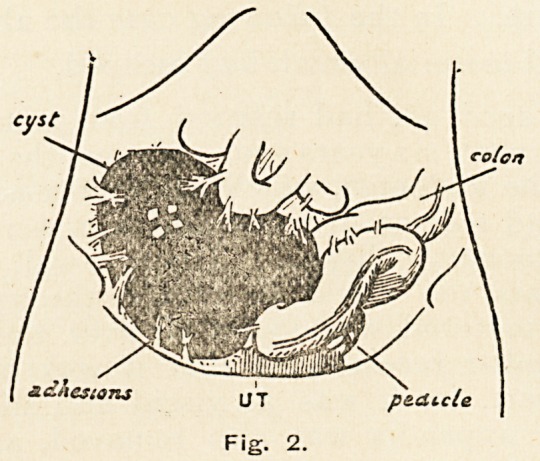# The Dangers of Delay in Ovariotomy

**Published:** 1903-12

**Authors:** T. Carwardine

**Affiliations:** Assistant-Surgeon to the Bristol Royal Infirmary.


### THE DANGERS OF DELAY IN OVARIOTOMY.

BY

T. Carwardine, M.S., M.B. (Lond.), F.R.C.S.,

*Assistant-Surgeon to the Bristol Royal Infirmary.*

---

It may be stated as a rule, to which there is no reasonable exception, that when a woman has an ovarian cyst she must either die or undergo an operation for its removal. Using the term ovariotomy in its wide and generally accepted sense as applicable to the removal of cystic tumours of the ovary and broad ligament of a sufficient size to cause symptoms complained of by the patient and signs which may be detected clinically, the arguments in favour of early operation are now convincing; whereas a few decades ago, when abdominal operations were associated with a very high mortality, ovariotomy too often meant immediate death.

Delay on the part of the patient is permissible, though unwise; but delay on the advice of a member of the profession is usually inexcusable, when once the diagnosis of an ovarian cyst is made. Delay also arises from erroneous diagnosis, particularly when the condition is mistaken for ascites or tuberculous peritonitis, but this aspect of the question needs only to be mentioned and passed over.

The question to be discussed here is this: once an ovarian cyst is diagnosed is it safe to defer operation? And I will illustrate the dangers of delay by reference to some cases which have been under my care.

1. **The size of the tumour** becomes in time an element of added danger. The increase in size is usually progressive, and an ovarian cyst may become dangerous from magnitude alone in from one to three years. In one case[1] the cyst contained 100 litres of fluid, and the pendulous abdomen reached down to the knees, the patient being only 25 years of age. In another remarkable case[2] the contents of the tumour weighed half as much again as the patient. The magnitude of the tumour gives rise to three classes of dangers:—

*Pressure effects* lead to œdema of back and limbs, to dilatation of the ureters, hydronephrosis and uræmia, to œdema and collapse of the lungs, to chronic intestinal obstruction and the like.

*The difficulty of operation* is increased. Not only is a larger incision required owing to the frequent presence of solid material or numerous minute cysts in the mother wall, but adhesions are commonly met with necessitating a more free exposure for their safe division.

[1] *Am. J. Obst.*, 1895, xxxi. 512. [2] *Med. Rev.* (St. Louis), 1893, xxviii. 3.

*Shock.*—It occasionally happens that when a large tumour is extruded from the abdomen, or a large cyst emptied of its contents, the patient becomes profoundly collapsed, particularly if there be much traction on the parts. I have met with one case in which this factor alone nearly cost the patient her life.

Mrs. H., aged 60, had suffered from indigestion, and twelve months previously had an attack of colic, when an uncertain cystic condition was noticed on the right side. A firm tumour reached to two inches above the umbilicus, and pressed upon a retroverted uterus below. The first stage of the operation was straightforward, but after extrusion of the cyst, and while the pedicle was being examined, the patient became profoundly collapsed. Artificial respiration was resorted to for ten minutes, and strychnine administered hypodermically: then the colour of the face, the pulse, and the normal character of respiration returned. She made a good recovery.

2. **Inflammation** is likely to occur in ovarian cysts from various causes; and, apart from torsion of the pedicle, the larger cysts are more liable to this complication.

*Peritonitis* of a simple plastic character is a rather common accompaniment, the patient having attacks of abdominal pain with pyrexia, causing her to take to bed. As a result of these attacks adhesions form. In rarer cases peritonitis is due to leakage or rupture; and if the cyst happen to be papilliferous, the peritoneum is likely to be grafted with the material, and a general papillomatous peritonitis results. The following is an example of repeated attacks of simple peritonitis.

E. H., a single woman, aged 27, had been subject to "wind attacks" during the last six months, and on two or three occasions had been confined to bed with pyrexia and abdominal pain, and had a severe attack of this character recently. The uterus was found to be retroverted, and a transverse cystic swelling occupied the lower half of the abdomen. On cœliotomy, the cyst was found adherent to the abdominal parietes, the pelvic wall, the omentum and intestines, and the contents were colloid and papillomatous. The cyst arose from the left broad ligament: its pedicle was twisted to the right for one whole turn, and when untwisted presented an anæmic circle at the site of torsion. The left ovary presented a cystic corpus luteum, which was incised, the ovary being preserved. Recovery.

*Suppuration* of an ovarian cyst is a serious complication, and renders the patient extremely ill, causes the cyst to become firmly adherent to contiguous parts, and ends in death unless relieved. Rarely a suppurating cyst has burst into the bladder, bowel, vagina, or through the parietes. Unless the suppuration be of recent onset, there will probably be no chance of separating the adhesions, and drainage will be the only treatment possible.

A. W., a married woman, aged 36, was seen in 1898 for a swelling in the pelvis the size of a hen's egg, and she was advised that an operation would be necessary, but she delayed. In the middle of 1902 she was seen in great pain, and was pale and emaciated, with a temperature of 103. An immediate operation was performed. A thin-walled cyst was found, adherent to and surrounded by coils of gut everywhere within reach, and the contents were foul and bloody. Two large drainage tubes were inserted, as well as three pieces of gauze each about two feet long. The temperature at once fell, and she went home convalescent a month afterwards. She returned in August, 1903, with a similar condition on the left side, though not so large as the former. This was drained through an opening in the left inguinal region, the cyst wall being quite thick and adherent, and enclosing about two pints of odourless pus. She again recovered.

*Fæcal infection* is a danger much more serious than mere suppuration, and it is a risk to which all fluid collections contiguous to the intestines are liable, and in the case of infection of a large ovarian cyst the symptoms soon assume a grave aspect. The following is a case of fæcal infection of a universally adherent cyst ending in death, and in this result differing from all the other cases, as well as being the only fatal case I have had.

E. M., aged 24, had noticed that for some months she had been getting big in the stomach. Four years previously she had what was called peritonitis, and was treated for six months in an institution by ointment applied to the abdomen. Three days ago she had severe abdominal pains of a sharp type, and has since had cold shivers on and off. She was a healthy-looking woman, with a temperature of 100 to 103, and presented a tender and rather fixed fluid abdominal tumour the size of a full-term uterus. The cervix was soft, moved with the tumour, and the fundus could not be separately made out, so that there was considerable difficulty in excluding the possibility of pregnancy. The patient was rather drowsy, and the tumour became larger and more tender during the succeeding twenty-four hours. On opening the abdomen a cyst was found firmly adherent to the parietal peritoneum. Several pints of puriform fluid were withdrawn, of a very strong fæcal odour, and containing masses and flakes of shreddy, cheesy material. The spine projected into the cyst behind, and the intestines bulged into it above. As a result of drainage the temperature at once fell, but the symptoms of fæcal poisoning continued, and progressive coma lasted till death. When subsequently examined, the cyst was found to have been perfectly drained and there were no signs of peritonitis. Intimate old adhesions existed everywhere—to the intestines generally, to the cæcum, to the sigmoid down to the rectum, and to the bladder, omentum, and parietes.

3. **The mobility** of a cyst arising from the ovary or broad ligament is a factor which may cause serious consequences, particularly when there is coincident pregnancy, as in two of the cases here recorded. The symptoms of torsion of the pedicle closely resemble those produced by intestinal obstruction, except that stercoraceous vomiting is unusual, and attacks of pain and vomiting accompanying a tender abdominal tumour are conspicuous features.

*Torsion.*—At first there is mere torsion, then venous obstruction occurs at the site of torsion. At this stage hemorrhage usually takes place into the cyst, which may be so great as to cause its rupture or the death of the patient. Finally, the arterial circulation is also arrested and the parts beyond the level of torsion become a blackened mass. This is usually described as gangrene, but, unless infected, the mass does not present the characters of moist gangrene and there is no stench; and as adhesions form during the process of strangulation, a certain amount of nutrition is provided by them. The following illustrates early torsion of an ovarian cyst complicating pregnancy.

Mrs. K., aged 31, consulted a surgeon in London 4½ years ago for a pelvic tumour, but operation was deferred because the tumour was not felt on a second examination. A fortnight ago she felt pain, tenderness, and a lump in the old spot. Amenorrhœa of three months corresponded to the signs of uterine pregnancy of that duration. A well-defined cystic tumour was felt in the right iliac region. This proved to be a dermoid of the right ovary the size of a large orange, the pedicle of which was twisted one-and-a-half turns to the left and was dark and congested. The cyst was removed entire, the patient made a good recovery, and the progress of pregnancy was not interrupted by the operation.

*Strangulation* is the effect of persistent torsion, and results in a necrotic appearance of the parts involved, which become swollen, succulent, and rotten.

Mrs. S. A. H., aged 42, complained of an abdominal tumour, increasing in size and accompanied during the last six weeks by labour-like attacks. The uterus was somewhat enlarged, raised, and soft, and a tense, elastic tumour occupied the pouch of Douglas; moreover, the uterus was myomatous. Next day the patient had retention of urine, and the uterus had become raised an inch higher into the abdomen. At the operation I found a tense, black, adherent cyst occupying the pelvis and lower abdomen. It arose from the broad ligament, was strangulated by torsion of 1½ turns to the right, and the corresponding ovary was enlarged, gangrenous, and rotten. The Fallopian tube was involved in the strangulated mass and spread out over its surface (Fig. 1).

Fig. 1.

The accompanying inflammation of the adjacent peritoneum may affect other parts also, causing their agglutination. Here is a case in which the pedicle was not only twisted, but was enclosed by a loop of adherent colon, the patient being also pregnant.

Mrs. S. had 1½ month's amenorrhœa, followed by two attacks of pain with an interval of a week between them, the pain being very severe in the lower abdomen, and passing into the thigh. A very tender abdominal swelling could be made out in front of the uterus, and for two or three inches above the pubes. Then there followed sudden increase of pain with collapse. Signs of peritonitis also became manifest, the swelling increased to the level of the umbilicus, the temperature rose, and the pulse became small and thready, 120. At the operation a dark bluish-black, soft, cystic mass presented, adherent to congested peritoneum in front, to the pelvic and abdominal parietes on the right, and to intestine on the left. The cyst was emptied, separated, and drawn out, revealing an enlarged uterus below. The pedicle passed through a hole formed by a loop of firmly adherent large intestine. On separating the adherent coils the pedicle appeared black, and rotated for two whole turns, and the pelvis contained some free fluid.

Fig. 2.

Some 36 hours afterwards she aborted a two months' fœtus, and eventually made a complete recovery (Fig. 2).

*Transplantation* of the tumour is another effect of torsion of its pedicle, and several cases have been recorded in which an ovarian cyst has become divorced from its original attachment, and has become yoked by fresh bonds to a distant part of the abdomen, even to the liver. When a fresh attachment obtains the original association is apt to be overlooked or forgotten.

4. **Intestinal obstruction** may be another result of delay in the removal of an ovarian cyst, and there are three ways in which this may arise:—

*From direct pressure.*—The mere pressure of a large cyst upon the intestines may be sufficient to cause a certain amount of chronic obstruction, but this will usually give way to saline aperients and enemata repeatedly administered.

*From adhesions.*—These may be sessile unions between the cyst and gut, interfering with the natural peristalsis or causing kinking of the bowel; or they may be band-like, and give rise to the same signs and varieties of obstruction as bands in general.

*From strangulation by the pedicle* of a cyst crossing the axis of the gut in a simple or complicated manner.

5. **Changes in the cyst wall.**—These comprise rupture, malignancy, and calcification.

*Rupture* may result from injury, strain, or over-distension, and may be repeated after the cyst has refilled. Thus I remember a woman who on two occasions emptied the contents of an ovarian cyst of large size into a chamber whilst in the squatting posture. In the following case the abdomen was full of viscid mucoid material which had escaped.

Mrs. S. P., aged 48, had suffered from metrorrhagia since her last confinement, six years ago. Six months ago she noticed a swelling in the abdomen, which has increased much of late. Albuminuria and œdema of trunk and legs were present, with some basal pulmonary collapse. The cyst wall was very thick, and the contents too viscid to escape through the trocar. Similar viscid material was found all over the abdomen, including the lumbar recesses, so that it was only possible to remove it in part, for it was as viscid and sticky as treacle. The vermiform appendix was also removed, and the patient recovered.

*Malignancy* of the wall of an ovarian cystoma, or of the matrix of a solid tumour, needs no argument for its early removal: the earlier such a tumour is removed the better for the patient and the surgeon, if removed at all.

*Calcification* of the wall is a rare secondary change which has occasionally been met with.

6. **Adhesions.**—These become very serious obstacles to the removal of old and large ovarian cysts, and even in the present day they are occasionally met with of so extensive a character as to defy the manipulative ingenuity of the surgeon in his endeavours to avoid damage to important structures, particularly the vascular supply to the intestine, and the ureter.

*The vermiform appendix* is not uncommonly involved, and may require removal—a comparatively simple matter.

*Intestinal obstruction* may result, as we have before described, from sessile or band-like adhesions.

*Other viscera* in the peritoneal cavity, any or all, may be involved and become a menace to successful operation, from immediate difficulties and remoter risks.

The following will serve as examples, one of the cases being of interest from the bearings of the influence of ovariotomy on cancer of the breast.

Mrs. S. P., aged 45, complained of severe abdominal pain, and of a tumour reaching above the umbilicus, firm, lobular, and dull on percussion. The uterus was long and low. Four years ago she had what was called a malignant cancer of the breast, and the nipple is now seen to be drawn into a deep fold; and $\frac{3}{4}$-inch to its outer side is a depressed, puckered scar, with a dry ulcer at the bottom having indurated edges, the induration extending a short way into the surrounding skin. The cyst was generally adherent by somewhat recent adhesions which bled easily, and the Fallopian tube and a very large pampinniform plexus of veins were removed also.

The case is of interest in the bearings of ovariotomy upon cancer of the breast, for when the patient left some three weeks afterwards the breast had distinctly improved. I regret that no microscopic specimen was taken to support this evidence and it may be noted also that the disease of the breast was on the opposite side to that of the ovary.

E. H., aged 41, was seen by me nine months previously for an ovarian tumour, but did not take the advice as to its removal. She noticed herself big three years ago, and was thought to be pregnant after three years of fruitless married life. A few months afterwards she had sickness and abdominal pains for nine days. At first there was amenorrhœa, but later the discharges were irregular and excessive. The uterus was found depressed, and a lobulated tumour occupied the whole abdomen. At the operation it was found that the tumour was chiefly solid and adherent, necessitating an incision from the xiphisternum to the pubes, and eventration after separation of adhesions. The adhesions were so intimate below that it became necessary to enucleate the mass as well as one possibly could, and to divide and ligate the strong attachments *seriatim*. It seemed at first as though it would be impossible to get the tumour away safely, and care had to be taken that the ureters were not injured. Two pints of normal saline fluid were left in the abdomen; and there was some shock afterwards and troublesome vomiting, but the patient made a rapid recovery and soon gained in weight.

7. **Ovarian tumours complicating pregnancy.**—It is only within recent years that the removal of tumours of the uterus and adnexa during pregnancy has been a recognised method of treatment, and I remember the trepidation with which certain London surgeons attempted the operation some years ago. It has fallen to my lot to perform the operation on three occasions with successful results. Two of the cases have been already referred to. In one case uncomplicated abortion resulted, and in the other two the pregnancy was uninterrupted, although, in one of them, operation extensively involved the pregnant uterus itself also. Under such circumstances there is, in my opinion, considerable danger in delaying the removal of an ovarian tumour because a woman is pregnant. Indeed, ovarian cysts become specially dangerous during the incubation of the fœtus in the womb, and the dangers concern the three stages of child-bearing.

*During pregnancy* for many months an ovarian tumour is accompanied with all the risks previously referred to, besides those to both mother and child, from its association with the enlarging uterus, and a special tendency to torsion and strangulation during the pregnant state.

*During labour* there are the risks of obstruction to delivery, the dangers of rupture, and the possibilities of infective changes.

*During the puerperium* there is increased danger from septic changes and from mechanical effects within the abdomen, particularly when an ovarian cyst has become adherent to some part distant from the involuted womb.

From the foregoing considerations, it is a reasonable rule of advice that, under all ordinary circumstances, an ovarian tumour should be removed as soon as diagnosed, and that delay is dangerous.

---

## Figures and Tables

**Fig. 1. f1:**
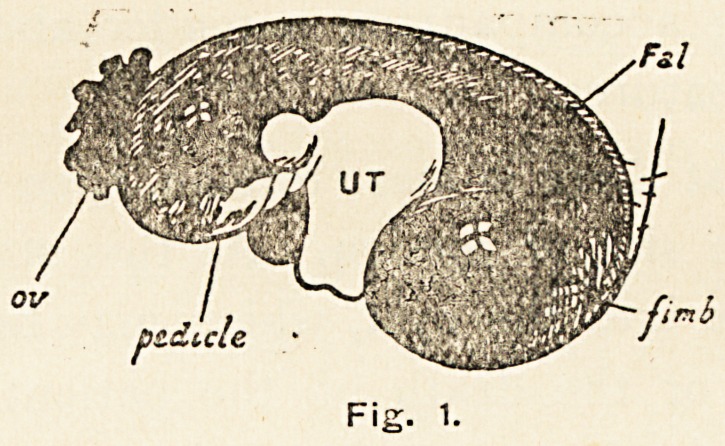


**Fig. 2. f2:**